# Tongue Sarcoidosis: A Rare Case Report and Review of the Literature

**DOI:** 10.1002/ccr3.72439

**Published:** 2026-04-06

**Authors:** Saede Atarbashi‐Moghadam, Leyla Roghanizadeh, Ali Lotfi, Mehrab Aghdasi, Mohammadreza Kashefi Baher

**Affiliations:** ^1^ Department of Oral and Maxillofacial Pathology, School of Dentistry Shahid Beheshti University of Medical Sciences Tehran Iran; ^2^ Iranian Center for Endodontic Research, Research Institute for Dental Sciences Shahid Beheshti University of Medical Sciences Tehran Iran; ^3^ Research Committee, School of Dentistry Shahid Beheshti University of Medical Sciences Tehran Iran; ^4^ Health Research Center Chamran Hospital Tehran Iran

**Keywords:** granulomatous, mouth, oral cavity, sarcoidosis, tongue

## Abstract

This study presents a case report with a literature review describing a rare manifestation of oral sarcoidosis involving the tongue in a 57‐year‐old woman with a prior diagnosis of cutaneous sarcoidosis. The lesion appeared as painless grayish‐white papules on the tip and dorsal surface of the tongue. A review of the reported cases of tongue sarcoidosis indicates lesions usually occur during the fifth and sixth decades of life, with a marked predominance in females. The most observed signs were swelling and nodules, which commonly affect the tip and dorsal surfaces, in contrast to the usual sites of oral squamous cell carcinoma. Various approaches have been described for managing oral sarcoidosis, ranging from observation without treatment to pharmacological (steroids/immunosuppressive) and surgical interventions. Since in many cases, oral lesions constitute the initial manifestation of sarcoidosis, as well as the wide variety of clinical symptoms and involvement of vital organs/systems (e.g., pulmonary, cardiac, lymph nodes) in the majority of sarcoidosis patients, dentists should regularly examine oral cavity, pay attention to the signs and symptoms of the disease, obtain biopsies of lesions if suspicious, and refer the patient to the relevant specialist.

## Key Clinical Message

Lingual sarcoidosis is a rare yet clinically significant entity that may present as non‐tender papules or nodules on the dorsal surface or tip of the tongue, features that help distinguish it from squamous cell carcinoma. Early recognition may represent the initial manifestation of systemic involvement, facilitating timely referral and management.

## 1 Introduction

Sarcoidosis shows a systemic non‐caseating granulomatous disease with multiple organ involvement and unknown etiology [1, 2, 3, 4, 5, 6]. The most common presentation of head and neck sarcoidosis is asymptomatic swelling of the parotid glands or cervical nodes [5]. Clinical symptoms are related to ethnicity, chronicity, location, extent of the disease, and granuloma activity [1]. Genetics, environmental agents, and infection have been described as contributing factors [1, 3]. Genetic variations that increase susceptibility to sarcoidosis may be located at sites that affect the immune response. T‐helper 1 (Th1) lymphocytes play a critical role in granuloma formation, which appears to be the result of the deposition of poorly insoluble antigenic material in the tissue. Antigens are taken up by antigen‐presenting cells, such as macrophages or dendritic cells, and subsequently presented to T lymphocytes. Due to the secretion of chemokines and cytokines by Th1 cells, mononuclear phagocytes and other inflammatory cells migrate to the site of antigen deposition, which results in the formation of a granuloma [1].

Oral presentation of sarcoidosis is rare, and the clinical presentation is not well defined [2]. Most cases are solitary and show a non‐tender firm swelling or nodular lesion that may affect any site of the oral cavity [4, 7, 8]. However, multiple lesions have also been reported in the oral cavity [1, 4]. This condition is a diagnosis of exclusion, with biopsy serving as a key diagnostic tool. Not all patients require medical or surgical treatment, as management depends on the lesion's location and clinical behavior [1, 7, 9].

Given the existing lack of consensus regarding the typical clinical presentation and optimal management of oral sarcoidosis, particularly in uncommon manifestations such as papular lesions, this study presents a rare case of lingual sarcoidosis, detailing its clinical and histopathological features, along with a comprehensive review of previously reported cases to elucidate the clinicopathological characteristics and treatment modalities of the disease.

## 2 Case History/Examination

**FIGURE 1:**
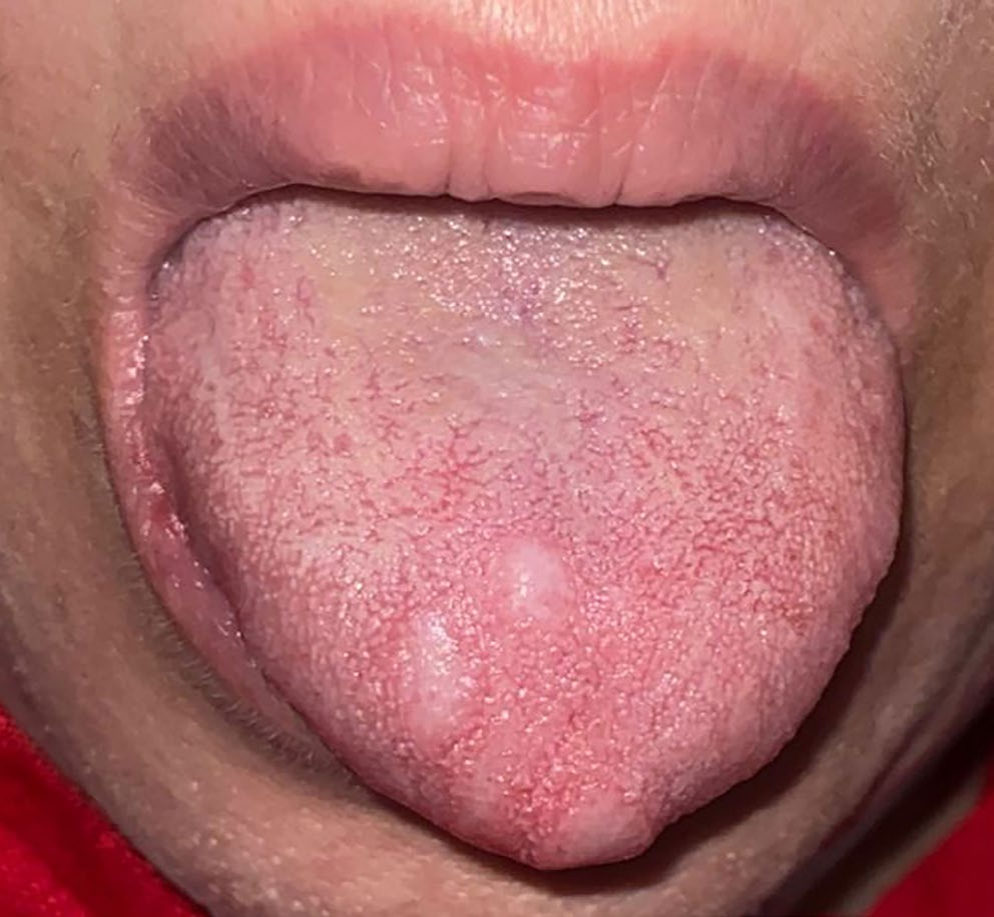
Multiple grayish‐white papules at the tip and dorsal surface of the tongue.

A 57‐year‐old female was referred to a private oral pathology center (Tehran, Iran) for evaluation of multiple non‐tender, firm, grayish‐white papules on the dorsal surface and tip of her tongue (Figure 1). Multiple erythematous papules were also noted around the nostrils and nasal tip. The patient's medical history revealed skin lesions around the nose, which were confirmed to have a facial cutaneous sarcoidosis pattern by biopsy with the characteristics of lupus pernio (LP). No evidence of systemic sarcoidosis involving other organs (lungs, heart, etc.) was observed. Under dermatologic supervision, the patient received methotrexate for 8 years, which was discontinued following the development of drug‐induced liver injury.

**FIGURE 2:**
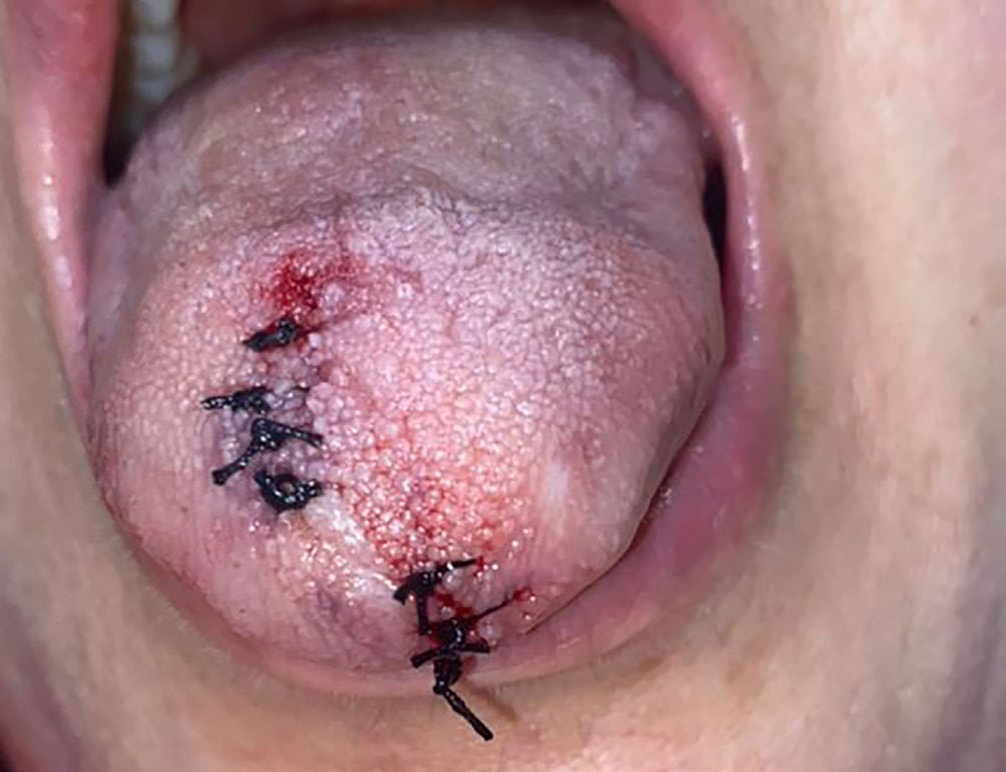
Excisional biopsy of tongue papules.

**FIGURE 3:**
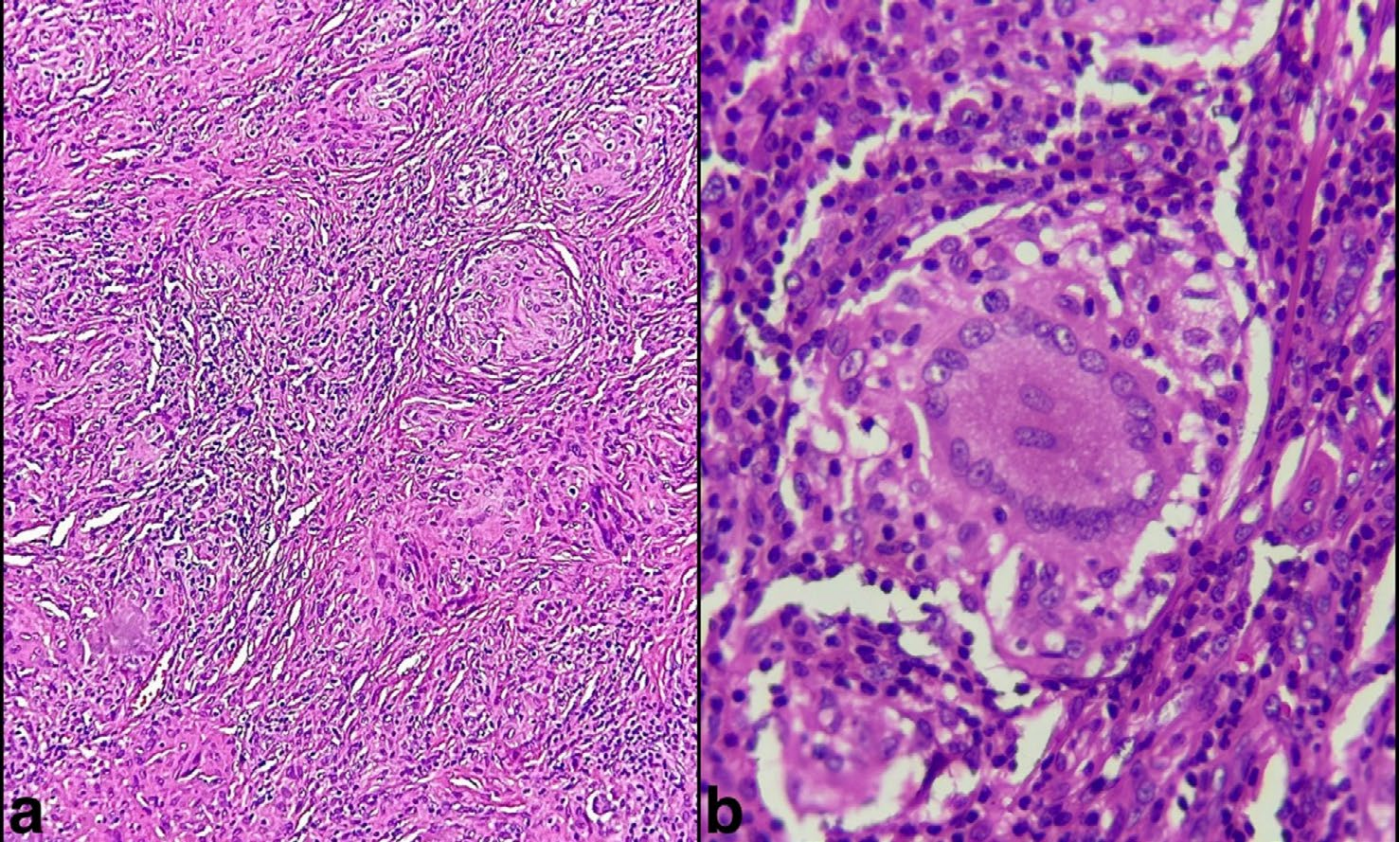
(a) Section showing well‐formed non‐caseating granulomatous inflammation composed of histiocytes, lymphocytes, and multinucleated giant cells (H and E, 100×). (b) High‐power view showing a large multinucleated giant cell (H and E, 400×).

The excisional biopsy of the tongue lesions was performed with a provisional clinical diagnosis of oral sarcoidosis (Figure 2). The microscopic examination showed tightly clustered aggregates of non‐caseating granulomatous inflammation composed of histiocytes, lymphocytes, and multinucleated giant cells (Figure 3a,b). No foreign body material was detected, and staining results were negative for fungal infection. Considering previous history and histopathological findings, a diagnosis of oral sarcoidosis was confirmed.

## 3 Differential Diagnosis, Investigations, and Treatment

The clinical differential diagnoses included oral sarcoidosis, multiple focal fibrous hyperplasia, and benign soft‐tissue tumors. However, based on the patient's history, oral sarcoidosis was considered the most likely diagnosis. Additionally, the histopathological differential diagnoses comprised sarcoidosis, foreign‐body granulomatosis, and fungal granulomatosis.

Investigations comprised a comprehensive medical and medication history taking, along with clinical and paraclinical examinations. Detailed intra‐ and extraoral examinations were performed. Laboratory investigations demonstrated elevated angiotensin‐converting enzyme (ACE) levels and the presence of diabetes mellitus. In addition, the patient reported that a chest computed tomography (CT) scan performed prior to referral to our center at the dermatologist's request was unremarkable and showed no evidence of bilateral hilar lymphadenopathy (BHL). Histopathological examination confirmed the diagnosis of sarcoidosis.

Overall, the present case exhibited a prolonged therapeutic course for oral and cutaneous involvement. Cutaneous involvement was addressed with various systemic medications, including prior methotrexate treatment and ongoing therapy with prednisolone (5 mg every other day), tofacitinib (5 mg daily), and leflunomide (10 mg daily). Furthermore, the lingual lesion underwent surgical excisional biopsy for definitive diagnosis and local management. Although systemic medications were primarily prescribed for the management of cutaneous lesions, their potential systemic effects cannot be overlooked and may have contributed to the therapeutic response of the oral lesions outlines. Table S1 the chronological course of the disease and the corresponding therapeutic interventions.

## 4 Outcome and Follow‐Up

Over a 3‐year follow‐up period, no involvement of organs other than the skin and tongue was identified. The skin lesions demonstrated partial remission, leading to dose tapering of systemic medications. However, subsequent lesion progression with an intermittent waxing and waning course following tapering necessitated reinstatement of the original dosing regimen. In addition, although surgical excision successfully resolved the existing lingual papules, new similar lesions emerged at sites distinct from the previously treated areas.

## 5 Discussion

This study reports an intraoral manifestation of sarcoidosis involving the tongue, characterized by non‐tender papules with granulomatous inflammation, occurring years after the initial diagnosis, treatment, and clinical remission of cutaneous sarcoidosis. Furthermore, to identify all documented cases of oral sarcoidosis involving the tongue, a comprehensive literature review was performed. We conducted a literature search of online databases (e.g., PubMed, Scopus, and Web of Science) using the keywords (“Sarcoidosis”) AND (“Tongue” OR “Lingual” OR “Oral”) with the time limitation from 1980 to July 2025. The literature selection process followed a PRISMA‐style screening framework; however, it was not conducted as a formal systematic review. We selected eligible case reports and case series from the search results. The inclusion criteria were English‐language cases presenting oral sarcoidosis confirmed by biopsy that must also have had tongue lesions. Cases were included based on the agreement of the independent reviewers, who assessed the titles, abstracts, and full‐text papers. After screening, 21 cases from 17 publications, plus the present case, were included for further analysis. The literature search was completed on December 10, 2025. Demographic, clinical, and management details of the reported tongue sarcoidosis cases have been summarized in Table 1.

**TABLE 1:** Demographic, clinical, and management details of tongue sarcoidosis cases.

First Author/Year	Age (Years)/Sex	Intraoral involvement location	Color	Clinical findings	Oral lesion as initial manifestation	Extraoral involvement location	Management/Outcome	Follow‐up (Years)
Van Maarsseveen/1982 [8]	69/F	Tongue (tip)	NA	Nodule	Yes	Lung	Surgery for oral lesion/Resolved (no other treatment)	3
Macleod/1985 [22]	50/F	Tongue (dorsal side)	NA	Swelling	Yes	None	Steroid/Resolved	1
Mendelsohn/1992 [20]	43/M	Tongue (lateral and ventral side)	NA	Swelling	Yes	BHL	No treatment/No change thereafter	1.5
Soto/1997 [7]	56/F	Tongue (ventral side), Buccal mucosa	Red (erosive)	Papule	No	Lung	Steroid/Marked remission	NA
Nagata/1999 [5]	32/F	Tongue (tip)	Brownish red	Nodule	Yes	BHL, Skin (LPand EN), Mediastinal lymphadenopathy	(1) No treatment for oral lesions/No change thereafter (2) Steroid injection in the LP lesion	NA
Simon/2003 [23]	41/M	Tongue (tip)	NA	Swelling	Yes	None	NA	NA
Soumithran/2005 [11]	40/F	Tongue (dorsal and ventral side), Floor of the mouth	Grayish white	Swelling	Synchronous	Skin (LP)	NA	NA
Koike/2007 [17]	48/M	Tongue (dorsal side)	NA	Swelling	Yes	BHL, Ocular, Spleen	Steroid/Resolved	1
Marie/2008 [13]	25/F	Tongue (tip)	Red (yellowish center)	Ulcer	Yes	BHL, Liver (hepatomegaly)	Steroid/Resolved	NA
Poate/2008 [9]	33/F	Tongue (lateral side)	NA	Swelling	Yes	BHL, Trigeminal ganglion	Surgery for oral lesion, Steroid/Resolved	0.5
Marcoval/2010 [12]	53/F	Tongue (tip) Lip	Yellowish brown	Plaque, Nodule	Synchronous	BHL, Skin (LP), Mediastinal lymphadenopathy	Hydroxychloroquine/Partial remission	NA
Guilabert/2011 [14]	46/F	Tongue (dorsal side)	NA	Tender Nodule	Yes	None	(1) Cyclosporine/Resolved (2) Steroids/Relapsed (3) Methotrexate, Etanercept, and surgery/Not effective	1
Pitti/2012 [24]	40/M	Tongue (dorsal side)	Red (erythematous)	Nodule	No	Lung	No Treatment	NA
Bouaziz/2012 [4]	24/F	Tongue	NA	Bullous	Yes	Lung, Mediastinal lymphadenopathy	(1) Combined steroid and doxycycline therapy/No change thereafter (2) Combined steroid and hydroxychloroquine therapy/Relapsed	2
	75/M	Tongue	NA	Nodule	Yes	Cervical lymphadenopathy, Submandibular gland	Surgery/Resolved	4
	42/F	Tongue	NA	Ulcer	Yes	Mediastinal lymphadenopathy	Steroid/Resolved	1
	48/F	Tongue	NA	Nodule	Yes	Mediastinal lymphadenopathy	No treatment/Resolved	12
Mukku/2013 [21]	56/F	Tongue	NA	NA	No	Mediastinal lymphadenopathy, Lung	Steroid/NA	NA
Nico/2016 [2]	50/M	Tongue	NA	Nodule	NA	Mediastinal lymphadenopathy, Skin (LP)	NA	NA
	60/F	Tongue (tip), Lip	NA	Nodule	NA	Mediastinal lymphadenopathy, Skin (LP), Lung	NA	NA
Sato/2018 [25]	46/F	Tongue (dorsal side)	NA	Swelling	Yes	Skin	No Treatment	NA
Present case	57/F	Tongue (tip and dorsal side)	Grayish white	Papule	No	Skin (LP)	(1) Methotrexate, prednisolone, tofacitinib, and leflunomide for skin lesion/Partial remission (2) Surgery for oral lesions/Partial remission	3

Abbreviations: BHL, bilateral hilar lymphadenopathy; EN, erythema nodosum; F, female; LP, lupus pernio, M, male, NA, not available.

The reviewed cases of lingual sarcoidosis showed a marked female predominance (female: male ratio = 2.67:1) and most frequently affected individuals in the fifth and sixth decades of life, with a mean age of 47 ± 12.66 years. This aligns with the established epidemiologic pattern of sarcoidosis, which demonstrates a female predilection and a bimodal age distribution peaking at 25–35 and 45–65 years, irrespective of oral involvement [1, 3, 5, 7], and is consistent with the present case.

Sarcoidosis can affect different regions of the tongue, most commonly the tip and dorsal surface, distinguishing it from oral squamous cell carcinoma (OSCC), which predominantly involves the lateral and ventral surfaces [10].

Simultaneous intraoral involvement occurs alongside lingual lesions reported in the buccal mucosa [7], floor of the mouth [11], and lips [2, 12]. The lesions exhibited a range of colors, including brownish‐red, grayish‐white, yellowish‐brown, and red. The most frequently reported manifestations of lingual involvement were swellings and nodules. However, other presentations, including papules (as seen in the present case) [7], plaques [12], bullae [4], and ulcers [4, 13], have also been described. Additionally, distinct cases have demonstrated facial numbness [9], tenderness of the lesions [14], and macroglossia [4]. Overall, oral presentations of sarcoidosis are uncommon and have usually been designated as nontender, well‐circumscribed, brownish‐red or violaceous nodules or papules that infrequently reveal surface ulceration [7]. In contrast, the present case exhibited grayish‐white lingual papules. Moreover, according to the study by Nico et al. [2] cutaneous involvement is typically characterized by well‐defined papules, plaques, and nodules, which often resemble oral mucosal lesions. In the present case, similar features were observed, as both cutaneous and oral lesions were papular in nature, although they differed in color. Notably, less common cutaneous manifestations include ulcerative, lichenoid, atrophic, psoriasiform, ichthyosiform, and vitiligo‐like patterns.

Of the 22 cases reviewed in this study, in 20, data regarding the presence of concomitant systemic sarcoidosis and the order of occurrence of oral manifestations and systemic signs were available. In most of these cases (70%), oral lesions were the initial manifestation of sarcoidosis, appearing before the onset of systemic signs; in 10%, intraoral and systemic manifestations occurred simultaneously; and in 20%, oral involvement developed after systemic presentations. Conversely, Nagata et al. [5] reported that in most instances, the diagnosis of sarcoidosis precedes the appearance of clinically detectable oral manifestations. However, Marie et al. [13] recommended that a thorough oral examination be routinely performed in all patients with sarcoidosis. Owing to its dynamic environment and high cellular turnover, the oral mucosa is susceptible to a wide spectrum of systemic disorders, including hematologic, endocrine, immunologic, and nutritional conditions [15, 16]. This underscores the pivotal role of dentists and oral medicine specialists as first‐line identifiers of systemic disease through meticulous examination of the entire oral mucosa, enabling the detection of subtle abnormalities in both patients with known systemic conditions and those attending routine dental visits, thereby facilitating early diagnosis, timely referral, and appropriate patient management.

Sarcoidosis, irrespective of lingual involvement, most commonly presents with BHL, pulmonary infiltrates, and cutaneous and/or ocular manifestations [7]. Additionally, most reported cases of lingual sarcoidosis were associated with extraoral/systemic involvement (86.36%), most commonly affecting the lungs, hilar and mediastinal lymph nodes, and skin. Involvement of the eye [17], spleen [17], liver [13], cervical lymph nodes [4], submandibular gland [4], and the trigeminal ganglion [9] has also been reported. This pattern emphasizes the systemic nature of this granulomatous disease. Consistent with this observation, Guilabert et al. [14] proposed that even in isolated oral sarcoidosis, periodic evaluation for potential involvement of other organs is advisable.

Histopathological examination of a sarcoidosis lesion shows tightly clustered aggregates of epithelioid histiocytes with a surrounding rim of lymphocytes. Scattered giant cells, either of the Langhans or foreign body type, are evident. Schaumann and asteroid bodies may be seen. Special stains for fungal and bacterial organisms are negative. There is no evidence of a foreign body [1]. Bacterial, fungal, and viral infections, such as tuberculosis, syphilis, histoplasmosis, and actinomycosis, should be mentioned in the microscopic differential diagnosis [7]. Other possibilities comprise foreign body reactions and orofacial granulomatosis, such as Crohn's disease, granulomatous cheilitis, and Melkersson's syndrome [7, 14, 17]. Moreover, tuberculoid leprosy, syphilis, and leishmaniasis should also be considered [5]. Notably, serum levels of ACE and lysozyme are assumed to be indicative of sarcoidosis [17].

Corticosteroids have long been the first‐line therapy for systemic sarcoidosis, with methotrexate being the most frequently recommended second‐line agent. Azathioprine and leflunomide may also be prescribed in selected cases [18, 19]. A wide range of approaches has been described for managing oral lesions, spanning from no treatment to pharmacological and surgical treatments (alone or in combination). Among the reviewed cases, five patients received no treatment; the lesion resolved spontaneously in one case [4], while it remained unchanged in two others [5, 20]. Additionally, surgical intervention, including the current case, was performed in five cases [4, 8, 9, 14]. Moreover, eight cases received steroid therapy, either alone or in combination with another medication, and one case underwent concurrent surgery. Relapse occurred in two patients receiving steroids [4, 14]; in one of them, surgery also failed, but ultimately, the administration of cyclosporine led to successful control of the disease [14]. In the present case, surgical excision of lingual lesions, potentially enhanced by synergistic effects of systemic medications for cutaneous disease, led to the resolution of existing papules. However, the subsequent emergence of new lesions at sites distinct from the original excision sites suggests a refractory disease behavior. Furthermore, the patient declined further surgical treatment for oral involvement, prioritizing management of the persistent facial cutaneous lesions due to their impact on appearance and self‐confidence, and because she experienced no pain or functional impairment from the oral lesions. Moreover, most reported cases (75%) of tongue sarcoidosis treated with surgical excision appeared to demonstrate lesion resolution. Overall, the choice of treatment for oral sarcoidosis may depend on lesion behavior and the presence of concurrent extraoral involvement requiring a systemic therapeutic approach.

Although the precise cause of the disease remains uncertain, one reported case of lingual involvement was linked to beryllium exposure, suggesting that hypersensitivity to the metal may trigger granulomatous changes [21]. Since sarcoidosis is generally a rare disease, the heterogeneity of signs/symptoms and disease course, and lack of prognosis biomarkers make it difficult to conduct clinical trials on the treatment of patients, and as a result, there is no comprehensive clinical guideline for the management of sarcoidosis. Further long‐term longitudinal studies are needed to establish evidence‐based guidelines for the management of oral sarcoidosis and to enhance understanding of its underlying pathogenesis.

In conclusion, this case highlights lingual sarcoidosis in a patient with known cutaneous involvement, underscoring the clinical significance of this rare entity as a potential early indicator of systemic disease and emphasizing the need for prompt histopathological evaluation to ensure accurate diagnosis and timely multidisciplinary management.

## Author Contributions

**Saede Atarbashi‐Moghadam:** conceptualization, investigation, methodology, supervision, writing – review and editing. **Leyla Roghanizadeh:** data curation, formal analysis, investigation, methodology, supervision, writing – original draft, writing – review and editing. **Ali Lotfi:** project administration, supervision, validation, visualization. **Mehrab Aghdasi:** supervision, validation. **Mohammadreza Kashefi Baher:** conceptualization, data curation, formal analysis, investigation, methodology, project administration, writing – review and editing.

## Funding

The authors have nothing to report.

## Ethics Statement

The authors have nothing to report.

## Consent

Written informed consent for the publication of this case, including all demographic/clinical details and any accompanying photographs, was obtained from the patient.

## Conflicts of Interest

The authors declare no conflicts of interest.

## Supporting information


**Table S1:** Summary of the patient's disease course and treatment timeline.

## Data Availability

No dataset was generated during the report of the case. The data that support the findings of the “Review of Literature” are available from the corresponding author upon reasonable request.
