# Supplementary material for: Tongue Sarcoidosis: A Rare Case Report and Review of the Literature
Source: Clin Case Rep. 2026 Apr 6;14(4):e72439. doi: 10.1002/ccr3.72439 (PMC13052223; doi:10.1002/ccr3.72439)
Supplement: Supplementary file 1 — Table S1: Summary of the patient's disease course and treatment timeline. [file CCR3-14-e72439-s001.docx]

| **Supplementary Table 1. Summary of the patient’s disease course and treatment timeline** | | | |
| --- | --- | --- | --- |
| **Investigation/Treatment** | **Classification** | **Start Date** | **Clinical Indication and Rationale** |
| *Dermatology referral for development of perinasal erythematous lesions consistent with a lupus pernio pattern in 2014* | | | |
| **Methotrexate** | Immunosuppressive antimetabolite | 2014 | Initiated for long-term control of cutaneous sarcoidosis; resulted in partial remission but was discontinued due to drug-induced liver injury |
| *Referral to an oral pathology center for evaluation of lingual involvement in November 2022* | | | |
| **Excision biopsy** | Surgery | November 2022 | Performed for definitive diagnosis and local management of lingual lesions |
| **Prednisolone** | Systemic corticosteroid | January 2023 | Prescribed for persistent cutaneous involvement; treatment discontinuation resulted in lesion progression, necessitating ongoing therapy |
| **Tofacitinib** | Janus kinase (JAK) inhibitor | May 2023 | Added as combination therapy for persistent cutaneous involvement; treatment discontinuation resulted in lesion progression, necessitating ongoing therapy |
| **Leflunomide** | Immunomodulatory antirheumatic drug | May 2023 | Added as combination therapy for persistent cutaneous involvement; treatment discontinuation resulted in lesion progression, necessitating ongoing therapy |
| *Three-year follow-up demonstrated partial remission with an intermittent waxing and waning course of the cutaneous lesions, accompanied by the emergence of new lingual lesions at locations other than the previously excised sites* | | | |
| *Although medications were prescribed primarily for the management of cutaneous lesions under dermatologic supervision, their systemic effects cannot be disregarded and may also have influenced the clinical course of the lingual lesions. | | | |
